# Evaluating the Relationship Between Inertia Levels and Tendency to Medical Errors Among Nurses in Paediatric Clinics

**DOI:** 10.1111/jep.70069

**Published:** 2025-04-01

**Authors:** Veysel Can, Mehmet Bulduk, Abdullah Adıyaman

**Affiliations:** ^1^ Department of Nursing, Faculty of Health Sciences Van Yüzüncü Yıl University Van Turkey; ^2^ Department of Nursing, Faculty of Health Sciences Erzurum Technical University Erzurum Turkey

**Keywords:** inertia, medical error tendency nurse, paediatric clinics

## Abstract

**Objective:**

This study aimed to evaluate the relationship between the inertia levels of nurses working in paediatric clinics and their tendency to make medical errors.

**Methods:**

This descriptive study was conducted between April 2023 and June 2023 with nurses working in the paediatric clinics of a training and research hospital in a province in eastern Turkey. The Descriptive Information Form, Inertia Scale (IS) and Nursing Tendency to Medical Errors Scale (NTMSE) were used as data collection tools. The entire population was targeted without using a sampling method, and the study was completed with 221 nurses.

**Results:**

Of the nurses, 52.9% were between 26 and 33 years of age, and 52.0% were female. Additionally, 66.1% were single, 50.7% had an income less than their expenses, and 77.8% held a bachelor's degree. Inertia was significantly associated with age, marital status, income level, professional experience, duration of work and medical error training, while it was found to be lower among nurses who followed scientific publications (*p* < 0.05). However, there was no statistical correlation between the mean total score of the NTMSE and the descriptive characteristics (*p* > 0.05). An association was found between age and the falls subscale of the NTMSE, while nurses working in paediatric units exhibited higher malpractice tendencies in the falls, patient monitoring, and material safety subscales (*p* < 0.05). No statistically significant correlation was found between the IS and NTMSE (*p* > 0.05).

**Conclusion:**

The study determined that the inertia levels of nurses working in paediatric clinics were moderate and their tendency to make medical errors was low. Regular training programmes and professional development activities should be planned to reduce inertia levels and enhance professional performance. Additionally, improving the working conditions of nurses and strengthening supportive monitoring mechanisms are essential to prevent medical errors.

## 1 Introduction

Individual and environmental factors are stated to create negative effects on employees, such as boredom, laziness and fatigue, which are referred to as ‘inertia’ in the literature [1, 2, 3]. Over time, the definition of clinical inertia has become unclear in the literature, with terms like ‘therapeutic inertia’, ‘physician inertia’, ‘failure to act’ and ‘inactivity’ often being misinterpreted or used interchangeably [4]. Inertia has been defined as an individual's failure to act or remain inactive despite knowing what needs to be done, how to do it and the consequences of their actions. This has been emphasized as a significant factor leading to serious problems and productivity loss in professional life [5]. Particularly in the public sector, employees' tendencies toward inactivity, stagnation and failure to fulfil their duties have been associated with inertia, which is emphasized to weaken organizational commitment [6, 7, 8, 9].

Inertia among healthcare professionals leads to delays in necessary treatment adjustments [10], prolonged decision‐making in managing chronic diseases [11], complications due to overtreatment and worsened patient outcomes resulting from non‐adherence to guidelines [12]. Studies show that inertia negatively affects disease management and patient outcomes in chronic conditions, including chronic kidney disease, heart failure, chronic obstructive pulmonary disease, rheumatoid arthritis and multiple sclerosis [13, 14, 15, 16, 17, 18]. Moreover, inertia should not be limited to the failure to advance therapy when needed but should be viewed as part of a broader phenomenon [19]. Factors contributing to inertia can be categorized into three main groups: structural deficiencies in the healthcare system, issues of knowledge and trust in clinical processes and patient‐related factors such as lack of awareness and low health literacy [16].

In the context of paediatric nursing, inertia may contribute to malpractice risks. Malpractice, defined as a lack of knowledge or skills, medication errors and inadequate care, can have more severe consequences for paediatric patients [20, 21]. Factors such as early discharge, nurse shortages and rapid technological advancements increase the risk of malpractice in paediatric nursing [22]. It has been noted that improving education levels and communication skills reduces the risk of medical errors [20, 23]. Additionally, the combination of inertia with burnout and fatigue poses even greater risks to patient safety [6]. Addressing inertia through targeted interventions could improve both patient safety and care quality in paediatric nursing.

### 1.1 Aim

The literature highlights that inertia has become a significant issue in the workplace. This study aims to determine the inertia level and malpractice tendencies of nurses working in paediatric services, identify the socio‐demographic factors influencing inertia and malpractice and evaluate the relationship between inertia and the tendency to commit medical errors.

## 2 Methods

### 2.1 Design

This study was conducted in descriptive type.

### 2.2 Study Population and Sample Size

This descriptive study was conducted between April 2023 and June 2023 in the paediatric clinics (paediatric wards, paediatric emergency services, paediatric intensive care units and neonatal intensive care units) of a tertiary care hospital located in eastern Turkey. According to information obtained from the hospital management, a total of 283 nurses are employed in the paediatric clinics. No sampling method was used in the study, and the entire population was targeted. At the end of the study, 239 nurses were interviewed; however, 18 nurses who did not consent to voluntary participation were excluded. Additionally, nurses who were on leave or medical report could not be included. Consequently, the study was completed with the participation of 221 nurses.

#### 2.2.1 Inclusion Criteria

- Nurses working in paediatric clinics.
- Nurses who voluntarily agreed to participate in the study.
- Nurses who had completed at least the minimum required nursing education level in Turkey, which is graduation from a health vocational high school.

#### 2.2.2 Exclusion Criteria

- Nurses working in other wards.
- Nurses who did not voluntarily agree to participate in the study.
- Nurses who were on leave or on medical report.

### 2.3 Data Collection

Data were collected through face‐to‐face interviews with nurses working in various paediatric settings (including paediatric wards, paediatric emergency services, paediatric intensive care units and neonatal intensive care units) at a hospital providing tertiary healthcare services in a province in eastern Turkey. Nurses completed a questionnaire form, which took ~10–15 min. Prior to filling out the questionnaire, nurses were informed about the study's purpose and methodology and assured that no personal data would be collected. Written and verbal consent was obtained from nurses who agreed to participate voluntarily.

### 2.4 Data Collection Tools

All nurses were attempted to be reached without sampling. The dependent variables are the inertia levels and medical error tendency levels of the nurses. The independent variables consist of the socio‐demographic characteristics obtained through the descriptive information form. To collect the data, the Introductory Information Form was used to determine the socio‐demographic characteristics, the Inertia Scale (IS) was used to measure the inertia levels of the nurses, and the Tendency to Medical Error Scale was used to assess the malpractice tendency levels.

#### 2.4.1 Introductory Information Form

In order to determine the socio‐demographic characteristics (age, gender, education, marital status, income level, years of working in the profession and in children's services, satisfaction with the profession, following scientific publications, membership to a professional organization, etc.), a questionnaire form consisting of 15 questions was used [7, 17, 20, 24, 25].

#### 2.4.2 Inertia Scale (IS)

The IS, developed by Liao et al. [8], reported a Cronbach's *α* coefficient of 0.75 for the Learning Inertia (LI) subscale and 0.72 for the Experience Inertia (EI) subscale in its initial validation study. Çankaya [9] later reported a Cronbach's *α* coefficient of 0.70 for the EI subscale, while Çankaya and Demirtaş [26] documented a Cronbach's *α* coefficient of 0.75 for the LKIA subscale [9, 26]. In the study conducted by Uyurdağ and Yıldırım [25], the overall Cronbach's *α* coefficient of the IS was calculated as 0.81, with subscale values of 0.68 for LKIA and 0.78 for EI [25]. In the present study, the Cronbach's *α* coefficient for the IS was determined to be 0.747, with 0.591 for the LKIA subscale and 0.734 for the EI subscale.

#### 2.4.3 Nursing Tendency to Medical Errors Scale (NTMES)

Developed by Özata and Altunkan [27], this scale includes a total of 49 items rated on a 5‐point Likert‐type scale, with responses ranging from ‘1—*never*’ to ‘5—*always*’. It is organized into five sub‐dimensions: Medication and Transfusion Practices (MTP), Nosocomial Infections (NI), Patient Monitoring and Material Safety (PMMS), Falls (F) and Communication (C). Scores on the scale can range from a minimum of 49 to a maximum of 245 points, with the option to calculate a mean score by dividing the total score by the number of items. A high score indicates a low tendency toward medical errors, while a low score signifies a high tendency toward medical errors. The Cronbach's *α* internal consistency coefficient for the scale was reported as 0.954, demonstrating high reliability. In this study, the Cronbach's *α* coefficient was found to be 0.955.

### 2.5 Ethical Approval

Ethical approval was obtained from the Non‐Interventional Clinical Research Ethics Committee (date 17.03.2023, number 2023/03‐03). Study permission was obtained from the Health Directorate of the province where the study was conducted. Throughout the research process, the World Medical Association's Declaration of Helsinki Ethical Principles for Medical Research Involving Human Subjects was adhered to.

### 2.6 Statistical Analysis

Data were analyzed using IBM SPSS Version 26. Compliance with normal distribution was assessed with the Kolmogorov–Smirnov and Shapiro–Wilk tests. For comparing normally distributed scale scores between paired groups, the independent two‐sample *t*‐test was employed. The Mann–Whitney *U* test was used for comparisons involving non‐normally distributed data. For comparing non‐normally distributed data across three or more groups, the Kruskal–Wallis test was applied, and multiple comparisons were examined using Dunn's test. Relationships between non‐normally distributed variables were analyzed with Spearman's *ρ* correlation coefficient. Results are presented as mean ± standard deviation, median (range: minimum–maximum) for quantitative data, and frequency and percentage for categorical data. The significance level was set at *p* < 0.05.

## 3 Results

In terms of demographic characteristics, 52.9% of the participants were between the ages of 26 and 33, and 52.0% were female. It was determined that 66.1% of the participants were single, 50.7% had income less than their expenses and 77.8% held a bachelor's degree. Among the nurses included in the study, 37.6% worked in paediatric wards, and 37.1% had been working in the profession for 6–9 years. Regarding the duration of working in any paediatric clinic, 48.0% of the participants had worked there for 1–2 years, 44.3% worked 41–63 h/week, and 84.6% worked both day and night shifts. When analyzing satisfaction with the profession, 54.8% of the participants were not satisfied. While 52% of the nurses participated in a training programme on medical errors, 75.7% of the participants found this training programme useful. It was determined that 53.8% of the participants followed scientific publications, and 36.7% were members of a professional organization (Table 1).

**Table 1:** Descriptive statistics of demographic characteristics (*n* = 221).

	Frequency (*n*)	Percentage (%)
Age		
18–25	82	37.1
26–33	117	52.9
34 and above	22	10.0
Gender		
Female	115	52.0
Male	106	48.0
Marital status		
Married	146	66.1
Single	72	32.5
Divorced	3	1.4
Income status		
Income less than expenditure	112	50.7
Income matches expenditure	60	27.1
Income more than expenditure	49	22.2
Education status		
High school	5	2.3
Pre‐bachelor's degree	18	8.1
Bachelor's degree	172	77.8
Postgraduate	26	11.8
Working clinic		
Paediatric wards	83	37.6
Neonatal intensive care unit	54	24.4
Paediatric emergency department	44	19.9
Paediatric intensive care unit	40	18.1
Duration of employment in the profession		
1–2 years	67	30.3
3–5 years	53	24.0
6–9 years	82	37.1
10 years and above	19	8.6
Duration of work in the paediatric wards		
1–2 years	106	48.0
3–5 years	67	30.3
6–9 years	41	18.5
10 years and above	7	3.2
Weekly working duration		
30–40 h	96	43.4
41–63 h	98	44.3
64 h and above	27	12.3
Working shift		
Day and night	187	84.6
Daytime	21	9.5
Night	13	5.9
Satisfaction with the profession		
Not satisfied	121	54.8
Unsure	59	26.7
Satisfied	41	18.5
Receiving a training programme on medical errors		
Yes	115	52.0
No	106	48.0
Thinking that these training programmes are useful^a^		
Yes	87	75.7
No	28	24.3
Following scientific publications related to the profession		
Yes	119	53.8
No	102	46.2
Membership in a professional organization		
Yes	81	36.7
No	140	63.3

a These data reflect responses from only the 115 participants who received training on medical errors.

The mean total score for the IS was 46.5 ± 6.8. The mean score for the LI subscale was 22.3 ± 3.9, while the mean score for the EI subscale was 24.2 ± 4.2. For the medical error tendency scale, the mean total score was 227.6 ± 18.0. The mean scores for its sub‐dimensions were as follows: MTP, 85.2 ± 6.4; NI, 55.7 ± 5.7; PMMS, 40.1 ± 4.8; F, 23.1 ± 2.5; and C, 23.5 ± 2.4. Additionally, no statistically significant correlations were found between the total and sub‐dimension scores of the medical error tendency scale and those of the IS (*p* > 0.05, Table 2).

**Table 2:** Descriptive statistics of the scales and examination of the relationship between scale scores.

	$\bar{X}$ ± SD Median (Min/Max)	LKIA 22.3 ± 3.9 22.0 (9.0–35.0)		EI 24.2 ± 4.2 25.0 (7.0–35.0)		IS 46.5 ± 6.8 47.0 (22.0–67.0)	
		*r*	*p*	*r*	*p*	*r*	*p*
MTP	85.2 ± 6.4 87.0 (48.0–90.0)	−0.039	0.559	−0.064	0.342	−0.102	0.133
NI	55.7 ± 5.7 57.0 (23.0–60.0)	0.017	0.799	−0.072	0.287	−0.049	0.467
PMMS	40.1 ± 4.8 41.0 (25.0–45.0)	0.067	0.322	0.055	0.419	0.044	0.518
F	23.1 ± 2.5 25.0 (14.0–25.0)	0.045	0.502	−0.030	0.660	−0.025	0.706
C	23.5 ± 2.4 25.0 (13.0–25.0)	−0.031	0.651	0.056	0.407	−0.003	0.969
NTMES	227.6 ± 18.0 231.0 (156.0–245.0)	0.014	0.840	−0.006	0.930	−0.026	0.705

Abbreviations: C, communication; EI, experience inertia; F, falls; IS, inertia scale; LKIA, learning/knowledge inertia; Max, maximum; Min, minimum; MTP, medication and transfusion practices; NI, nosocomial infections; NTMES, Nursing Tendency to Medical Error Scale; PMMS, patient monitoring and material safety; *r*, Spearman's *ρ* correlation coefficient; SD, standard deviation; X̄, mean.

There was a statistically significant difference between LI and age, with greater differences observed in the 26–33 and 34‐and‐above age groups (*p* < 0.05). A significant difference was also found between marital status and both LI and the total inertia score, with higher levels of both in married individuals (*p* < 0.05). Income status was significantly related to LI and the total inertia score, with higher levels of LI and total inertia observed in nurses with higher income status (*p* < 0.05). Nurses with 10 or more years of professional experience had significantly higher LI scores compared to other groups (*p* < 0.05). Additionally, EI was significantly higher in nurses working in paediatric wards for 3–5 years and 6 years or more (*p* < 0.05). Nurses who had received training on medical errors exhibited significantly higher EI (*p* < 0.05). Conversely, LI and total inertia scores were significantly lower in nurses who regularly followed scientific publications related to their profession (*p* < 0.05). No statistically significant differences were found between inertia and its sub‐dimensions with respect to variables such as gender, educational status, working shift, job satisfaction, weekly working hours, perceived usefulness of medical error education or membership in a professional organization (*p* > 0.05, Table 3).

**Table 3:** Evaluation of the relationship between descriptive characteristics and inertia.

	LKIA	EI	IS
	Median (Min–Max)	Median (Min–Max)	Median (Min–Max)
Age			
18–25	21.0 (15.0–30.0)^a^	24.0 (14.0–31.0)	45.0 (31.0–60.0)
26–33	23.0 (12.0–33.0)^b^	25.0 (7.0–35.0)	48.0 (22.0–65.0)
34 and above	23.0 (9.0–35.0)^b^	25.0 (16.0–32.0)	49.0 (26.0–67.0)
Test statistic	10.458	3.767	7.346
p**	0.005	0.152	0.051
Gender			
Female	22.0 (12.0–33.0)	25.0 (7.0–31.0)	46.0 (35.0–61.0)
Male	23.0 (9.0–35.0)	24.0 (9.0–35.0)	48.0 (22.0–67.0)
Test statistic	6606	6053.5	6539.5
p*	0.28	0.93	0.348
Marital status			
Married	23.5 (13.0–35.0)	25.0 (7.0–35.0)	49.0 (22.0–67.0)
Single	22.0 (9.0–30.0)	24.0 (14.0–31.0)	46.0 (26.0–60.0)
Test statistic	4051	4497.5	4248.5
p*	0.003	0.051	0.012
Income status			
Income less than expenditure	22.0 (9.0–33.0)^b^	25.0 (7.0–31.0)	47.0 (26.0–61.0)^b^
Income matches expenditure	22.0 (16.0–32.0)^b^	24.0 (14.0–35.0)	45.5 (31.0–65.0)^b^
Income more than expenditure	23.0 (13.0–35.0)^a^	25.0 (9.0–33.0)	49.0 (22.0–67.0)^a^
Test statistic	9.632	3.402	9.048
p**	0.008	0.183	0.011
Education status			
High school and pre‐bachelor's degree	22.0 (9.0–29.0)	25.0 (9.0–31.0)	48.0 (22.0–57.0)
Bachelor's degree	22.0 (12.0–35.0)	24.0 (7.0–35.0)	46.0 (31.0–67.0)
Postgraduate	23.0 (17.0–28.0)	25.0 (15.0–30.0)	48.0 (35.0–57.0)
Test statistic	0.272	1.678	0.996
p**	0.873	0.432	0.608
Duration of employment in the profession			
1–2 years	21.0 (16.0–30.0)^a^	23.0 (14.0–30.0)	46.0 (31.0–60.0)
3–5 years	23.0 (15.0–30.0)^a,b^	25.0 (14.0–30.0)	47.0 (31.0–58.0)
6–9 years	23.0 (12.0–33.0)^a,b^	25.0 (7.0–33.0)	48.0 (22.0–61.0)
10 years and above	23.0 (9.0–35.0)^b^	25.0 (16.0–35.0)	49.0 (26.0–67.0)
Test statistic	9.662	5.599	8.089
p**	0.022	0.133	0.051
Duration of work in the paediatric wards			
1–2 years	22.0 (13.0–33.0)	24.0 (7.0–31.0)^a^	46.0 (22.0–60.0)
3–5 years	23.0 (15.0–30.0)	25.0 (14.0–33.0)^b^	48.0 (31.0–61.0)
6 years and above	22.0 (9.0–35.0)	25.5 (15.0–35.0)^b^	47.0 (26.0–67.0)
Test statistic	2.508	10.014	6.395
p**	0.285	0.007	0.051
Weekly working duration			
40 h and below	23.0 (13.0–33.0)	25.0 (7.0–30.0)	48.0 (22.0–57.0)
40 h over	22.0 (9.0–35.0)	25.0 (14.0–35.0)	46.0 (26.0–67.0)
Test statistic	5205.500	5561.000	5213.500
p*	0.090	0.349	0.094
Working shift			
Daytime	23.0 (17.0–33.0)	24.0 (7.0–29.0)	48.0 (31.0–54.0)
Night	20.0 (19.0–30.0)	22.0 (19.0–30.0)	43.0 (40.0–60.0)
Day and night	22.0 (9.0–35.0)	25.0 (9.0–35.0)	47.0 (22.0–67.0)
Test statistic	3.938	3.249	3.553
p**	0.14	0.197	0.169
Satisfaction with the profession			
Satisfied	23.0 (12.0–30.0)	25.0 (14.0–31.0)	47.0 (31.0–61.0)
Unsure	22.0 (16.0–30.0)	24.0 (15.0–35.0)	46.0 (31.0–65.0)
Not satisfied	22.0 (9.0–35.0)	24.0 (7.0–33.0)	47.0 (22.0–67.0)
Test statistic	0.657	0.673	0.675
p**	0.72	0.714	0.714
Receiving a training programme on medical errors			
Yes	23.0 (12.0–35.0)	25.0 (14.0–33.0)	47.0 (31.0–67.0)
No	22.0 (9.0–33.0)	24.0 (7.0–35.0)	46.0 (22.0–65.0)
Test statistic	5698.5	5095.5	5261
p*	0.402	0.034	0.079
Thinking that these training programmes are useful			
Yes	23.0 (12.0–30.0)	25.0 (14.0–31.0)	47.0 (31.0–61.0)
No	22.0 (15.0–35.0)	25.0 (15.0–33.0)	48.5 (33.0–67.0)
Test statistic	1157.5	1347	−0.811
*p*	0.692	0.398	0.419
Following scientific publications related to the profession			
Yes	22.0 (12.0–30.0)	25.0 (14.0–35.0)	46.0 (31.0–65.0)
No	23.0 (9.0–35.0)	25.0 (7.0–33.0)	48.0 (22.0–67.0)
Test statistic	7587	6639	7241.5
p*	0.001	0.227	0.013
Membership in a professional organization			
Yes	21.0 (12.0–30.0)	24.0 (9.0–35.0)	47.0 (22.0–65.0)
No	23.0 (9.0–35.0)	25.0 (7.0–32.0)	47.0 (26.0–67.0)
Test statistic	6284.500	5965.500	6165.500
p*	0.178	0.517	0.279

*Note:* Nonparametric tests: ‘*Mann–Whitney *U* test, **Kruskal–Wallis test’, median (minimum–maximum).

Abbreviations: EI, experience inertia; IS, inertia scale; LKIA, learning/knowledge inertia.

^a,b^ There is no difference between groups with the same letter.

Statistical analysis revealed a significant relationship between age and falls, a sub‐dimension of the medical error tendency scale. Specifically, nurses aged 18–25 years demonstrated a higher tendency towards medical errors related to falls compared to nurses in other age groups (*p* < 0.05). Additionally, nurses working in paediatric wards showed higher tendencies for malpractice in both falls and PMMS, with these findings also being statistically significant (*p* < 0.05). However, no significant correlations were found between malpractice and variables such as gender, marital status, income status, length of time working in the profession and in paediatric clinics, weekly working hours, job satisfaction, training on medical errors and its perceived usefulness or engagement with scientific publications related to the profession (*p* > 0.05, Table 4).

**Table 4:** Evaluation of the relationship between descriptive characteristics and tendency to medical error.

	MTP	NI	PMMS	F	C	NTMES
	Median (Min–Max)	Median (Min–Max)	Median (Min–Max)	Median (Min–Max)	Median (Min–Max)	Median (Min–Max)
Age						
18–25	87.0 (52.0–90.0)	56.0 (31.0–60.0)	40.0 (25.0–45.0)	23.0 (14.0–25.0)^a^	24.0 (13.0–25.0)	230.0 (156.0–245.0)
26–33	88.0 (48.0–90.0)	58.0 (23.0–60.0)	42.0 (26.0–45.0)	25.0 (17.0–25.0)^b^	25.0 (16.0–25.0)	236.0 (164.0–245.0)
34 and above	87.0 (81.0–90.0)	58.0 (48.0–60.0)	42.5 (32.0–45.0)	24.5 (17.0–25.0)^a,b^	25.0 (19.0–25.0)	234.0 (197.0–245.0)
Test statistic	0.288	4.514	2.129	8.673	1.166	2.564
p**	0.866	0.105	0.345	0.013	0.558	0.277
Gender						
Female	87.0 (53.0–90.0)	57.0 (23.0–60.0)	41.0 (25.0–45.0)	25.0 (16.0–25.0)	25.0 (17.0–25.0)	231.0 (171.0–245.0)
Male	87.0 (48.0–90.0)	57.5 (28.0–60.0)	42.0 (27.0–45.0)	25.0 (14.0–25.0)	25.0 (13.0–25.0)	231.5 (156.0–245.0)
Test statistic	5733	5847.5	6110.5	6009.5	5688	5832.5
p*	0.435	0.593	0.974	0.845	0.351	0.58
Marital status						
Married	87.5 (48.0–90.0)	58.0 (28.0–60.0)	42.0 (26.0–45.0)	25.0 (17.0–25.0)	25.0 (16.0–25.0)	234.5 (164.0–245.0)
Single	87.0 (52.0–90.0)	57.0 (23.0–60.0)	41.0 (25.0–45.0)	25.0 (14.0–25.0)	25.0 (13.0–25.0)	231.0 (156.0–245.0)
Test statistic	5158.5	5090.5	5382.5	5153.5	5216	5292
p*	0.637	0.529	0.966	0.609	0.718	0.871
Income status						
Income less than expenditure	88.0 (52.0–90.0)	58.0 (23.0–60.0)	42.0 (30.0–45.0)	25.0 (16.0–25.0)	25.0 (15.0–25.0)	232.5 (156.0–245.0)
Income matches expenditure	87.0 (48.0–90.0)	57.0 (28.0–60.0)	40.0 (25.0–45.0)	24.0 (18.0–25.0)	25.0 (13.0–25.0)	230.5 (164.0–245.0)
Income more than expenditure	86.0 (71.0–90.0)	56.0 (41.0–60.0)	42.0 (27.0–45.0)	23.0 (14.0–25.0)	24.0 (14.0–25.0)	232.0 (171.0–245.0)
Test statistic	1.023	1.803	3.487	3.145	0.944	1.802
p**	0.599	0.406	0.175	0.207	0.624	0.406
Education status						
High school and pre‐bachelor's degree	86.0 (76.0–90.0)	56.0 (49.0–60.0)	39.0 (32.0–45.0)	25.0 (19.0–25.0)	24.0 (19.0–25.0)	226.0 (198.0–245.0)
Bachelor's degree	88.0 (48.0–90.0)	58.0 (23.0–60.0)	42.0 (25.0–45.0)	25.0 (14.0–25.0)	25.0 (13.0–25.0)	232.0 (156.0–245.0)
Postgraduate	86.0 (73.0–90.0)	56.0 (44.0–60.0)	41.0 (31.0–45.0)	23.0 (17.0–25.0)	24.0 (19.0–25.0)	232.5 (187.0–245.0)
Test statistic	0.6	0.38	1.708	1.743	0.521	0.631
p**	0.741	0.827	0.426	0.418	0.771	0.729
Working clinic						
Child wards	86.0 (74.0–90.0)	56.0 (31.0– 60.0)	40.0 (25.0–45.0)^a^	24.0 (14.0–25.0)^a^	24.0 (13.0–25.0)	228.0 (169.0–245.0)
Neonatal intensive care unit	86.0 (71.0–90.0)	57.5 (44.0–60.0)	41.0 (30.0–45.0)^a,b^	25.0 (19.0–25.0)^b^	25.0 (18.0–25.0)	231.0 (187.0–245.0)
Paediatric emergency department	89.0 (48.0–90.0)	59.0 (23.0–60.0)	43.0 (31.0–45.0)^b^	25.0 (17.0–25.0)^b^	25.0 (18.0–25.0)	239.5 (156.0–245.0)
Paediatric Intensive Care Unit	89.5 (62.0–90.0)	58.0 (45.0– 60.0)	43.0 (30.0–45.0)^b^	25.0 (17.0–25.0)^a,b^	25.0 (16.0–25.0)	237.5 (172.0–245.0)
Test statistic	6.013	2.989	12.785	12.564	9.184	8.1
p**	0.111	0.393	0.005	0.006	0.051	0.051
Duration of employment in the profession						
1–2 years	87.0 (52.0–90.0)	56.0 (31.0–60.0)	40.0 (25.0–45.0)	23.0 (14.0–25.0)	24.0 (13.0–25.0)	231.0 (156.0–245.0)
3–5 years	88.0 (53.0–90.0)	58.0 (23.0–60.0)	42.0 (26.0–45.0)	25.0 (17.0–25.0)	25.0 (15.0–25.0)	233.0 (171.0–245.0)
6–9 years	87.0 (48.0–90.0)	58.0 (28.0–60.0)	42.0 (29.0–45.0)	25.0 (18.0–25.0)	25.0 (16.0–25.0)	234.0 (164.0–245.0)
10 years and above	87.0 (81.0–90.0)	57.0 (48.0–60.0)	42.0 (32.0–45.0)	24.0 (17.0–25.0)	25.0 (19.0–25.0)	230.0 (197.0–245.0)
Test statistic	3.374	2.535	2.443	7.015	0.874	1.732
p**	0.337	0.469	0.486	0.071	0.832	0.63
Duration of work in the paediatric wards						
1–2 years	87.0 (52.0–90.0)	57.0 (23.0–60.0)	41.0 (25.0–45.0)	24.0 (14.0–25.0)	25.0 (13.0–25.0)	231.5 (156.0–245.0)
3–5 years	88.0 (72.0–90.0)	57.0 (34.0–60.0)	41.0 (26.0–45.0)	25.0 (17.0–25.0)	25.0 (15.0–25.0)	233.0 (184.0–245.0)
6 years and above	84.5 (48.0–90.0)	58.0 (28.0–60.0)	42.5 (30.0–45.0)	25.0 (18.0–25.0)	25.0 (16.0–25.0)	229.5 (164.0–245.0)
Test statistic	3.47	0.319	1.134	4.862	0.961	0.525
p**	0.176	0.853	0.567	0.088	0.619	0.769
Weekly working duration						
40 h and below	86.0 (73.0–90.0)	56.5 (44.0–60.0)	41.0 (25.0–45.0)	24.0 (16.0–25.0)	24.0 (15.0–25.0)	230.5 (185.0–245.0)
40 h over	88.0 (48.0–90.0)	58.0 (23.0–60.0)	42.0 (27.0–45.0)	25.0 (14.0–25.0)	25.0 (13.0–25.0)	233.0 (156.0–245.0)
Test statistic	5439.000	5509.500	5192.500	5357.000	5168.500	5222.000
p*	0.223	0.286	0.083	0.140	0.055	0.098
Working shift						
Daytime	88.0 (74.0–90.0)	58.0 (45.0–60.0)	42.0 (26.0–45.0)	25.0 (17.0–25.0)	24.0 (17.0–25.0)	238.0 (185.0–245.0)
Night	90.0 (62.0–90.0)	58.0 (45.0–60.0)	39.0 (29.0–45.0)	24.0 (16.0–25.0)	24.0 (15.0–25.0)	223.0 (172.0–245.0)
Day and night	87.0 (48.0–90.0)	57.0 (23.0–60.0)	41.0 (25.0–45.0)	25.0 (14.0–25.0)	25.0 (13.0–25.0)	231.0 (156.0–245.0)
Test statistic	1.552	0.444	0.413	1.922	2.059	0.319
p**	0.46	0.801	0.813	0.382	0.357	0.853
Satisfaction with the profession						
Satisfied	88.0 (75.0–90.0)	57.0 (41.0–60.0)	42.0 (27.0–45.0)	25.0 (14.0–25.0)	25.0 (14.0–25.0)	235.0 (171.0–245.0)
Unsure	86.0 (72.0–90.0)	57.0 (31.0–60.0)	40.0 (30.0–45.0)	24.0 (18.0–25.0)	24.0 (13.0–25.0)	227.0 (169.0–245.0)
Not satisfied	87.0 (48.0–90.0)	58.0 (23.0–60.0)	42.0 (25.0–45.0)	25.0 (17.0–25.0)	25.0 (16.0–25.0)	234.0 (156.0–245.0)
Test statistic	0.852	1.014	1.734	3.242	2.476	1.297
p**	0.653	0.602	0.42	0.198	0.29	0.523
Receiving a training programme on medical errors						
Yes	86.0 (48.0–90.0)	58.0 (23.0–60.0)	42.0 (25.0–45.0)	25.0 (17.0–25.0)	25.0 (15.0–25.0)	232.0 (156.0–245.0)
No	88.0 (71.0–90.0)	57.0 (31.0–60.0)	41.0 (27.0–45.0)	25.0 (14.0–25.0)	24.0 (13.0–25.0)	231.0 (169.0–245.0)
Test statistic	6095	6101.5	5743.5	6153.5	5811	6052.5
p*	1	0.989	0.453	0.894	0.516	0.929
Thinking that these training programmes are useful						
Yes	88.0 (52.0–90.0)	59.0 (23.0–60.0)	42.0 (26.0–45.0)	25.0 (17.0–25.0)	25.0 (15.0–25.0)	234.0 (156.0–245.0)
No	84.0 (48.0–90.0)	55.5 (28.0–60.0)	41.5 (25.0–45.0)	23.5 (17.0–25.0)	25.0 (18.0–25.0)	228.0 (164.0–245.0)
Test statistic	949.5	938	1143	1080.5	1238	989
*p*	0.073	0.061	0.619	0.331	0.885	0.135
Following scientific publications related to the profession						
Yes	88.0 (48.0–90.0)	58.0 (28.0–60.0)	42.0 (29.0–45.0)	25.0 (16.0–25.0)	25.0 (15.0–25.0)	234.0 (156.0–245.0)
No	87.0 (53.0–90.0)	57.0 (23.0–60.0)	41.0 (25.0–45.0)	24.0 (14.0–25.0)	24.0 (13.0–25.0)	231.0 (169.0–245.0)
Test statistic	5599	5732.5	5917	5443.5	5299	5558.5
p*	0.31	0.466	0.745	0.153	0.077	0.28
Membership in a professional organization						
Yes	87.0 (52.0–90.0)	57.0 (23.0–60.0)	42.0 (26.0–45.0)	25.0 (16.0–25.0)	25.0 (16.0–25.0)	232.0 (156.0–245.0)
No	87.0 (48.0–90.0)	57.0 (28.0–60.0)	41.0 (25.0–45.0)	24.0 (14.0–25.0)	25.0 (13.0–25.0)	231.0 (164.0–245.0)
Test statistic	5316.5	5579	5414	5506.5	6050.5	5580
p*	0.43	0.838	0.571	0.699	0.366	0.844

*Note:* Nonparametric tests: ‘*Mann–Whitney *U* test, **Kruskal–Wallis test’, median (minimum–maximum).

Abbreviations: C, communication; F, falls; MTP, medication and transfusion practices, NI, nosocomial infections; NTMES, Nursing Tendency to Medical Error Scale, PMMS, patient monitoring and material safety.

^a,b^No difference between groups with the same letter.

## 4 Discussion

This study aims to evaluate the relationship between inertia levels and the tendency for medical errors among nurses working in paediatric clinics, as well as to assess the socio‐demographic factors influencing these variables. Increased inertia levels can adversely affect both the individual and their organization due to decreased productivity and performance [6]. The findings of this study indicate that the nurses exhibited a moderate level of inertia based on the assessment results. Previous research also reported moderate levels of inertia among nurses [7, 25].

Medical errors have recently become a significant concern across ethical, legal, medical, educational and methodological fields both globally and nationally [28]. It has been noted that factors contributing to medical errors among nurses include deficiencies in documenting nursing care, non‐compliance with regulations, insufficient staffing, high work intensity and inexperience [29, 30, 31, 32]. High scores on the NTMES are indicative of a lower tendency towards medical errors [27]. The findings of this study have shown that the nurses demonstrated a low tendency for medical errors, as indicated by the NTMES. Previous literature also reports low tendencies towards medical errors among nurses [28, 30, 31, 32, 33].

The findings indicate that there is no relationship between IS and NTMES. Although Artero‐Lopez et al. [24] and Uyurdağ and Yildirim [25] suggested that a high workload increases inertia, Aydın Sayılan and Mert Boğa [31] found no relationship between workload and the tendency to make medical errors. However, other studies have indicated that inertia among nurses negatively impacts patient care [24, 34]. Despite the lack of a relationship between IS and NTMES, it is believed that intermediate levels of inertia in nurses may lead to an increased tendency to make medical errors if inertia is not addressed.

The study found that LI levels were higher in nurses aged 26–33 years and those aged 34 and older compared to nurses aged 18–25 years. This finding indicates that while there is no statistically significant relationship between age and the overall inertia score, there is a significant relationship between the learning sub‐dimension and age. Research has shown that as individuals age, they rely more on their experiences, which can increase inertia in learning and acquiring knowledge [5]. However, other studies suggest no relationship between age and inertia [25, 35]. This discrepancy may impact nurses' self‐development, institutional advancement and evidence‐based patient care as they age.

The findings of this study indicate that marital status is a significant factor influencing inertia, with married nurses demonstrating higher levels of inertia compared to their unmarried counterparts. Conversely, Uyurdağ and Yildirim [25] found no significant relationship between marital status and inertia. This result may be influenced by the increased work and home responsibilities of married nurses, which could contribute to higher inertia.

Additionally, while literature suggests that a lower economic level may increase inertia in disease management [36, 37], this study found that both LI and total inertia scores increased with higher income levels, and this was statistically significant. These findings suggest that high‐income nurses might experience more difficulty in learning latest information or deviating from current practices due to the comfort and security provided by their stable living conditions.

In the study conducted by Uyurdağ and Yildirim [25], a significant relationship was found between professional experience and LI. The study reported that as the length of time in the profession increased, so did LI, primarily due to accumulated experience. The findings aligned with existing literature, revealing a statistically significant relationship between professional experience and LI. Notably, the statistical significance between the duration of working in paediatric wards and EI supports the conclusion that increased experience contributes to higher LI among nurses. This suggests that as nurses gain experience, they may become less inclined to acquire new knowledge or enhance their existing skills.

The study also found that nurses who did not follow scientific publications related to their profession exhibited higher levels of inertia and LI compared to those who did, with statistical significance. These results were consistent with the findings of Uyurdağ and Yildirim [25], which also indicated that inertia levels were higher among nurses who did not engage with scientific literature.

The study found that younger nurses showed a higher tendency for medical errors in the falls sub‐dimension compared to other age groups. Külcü and Yiğit [38] reported no relationship between age and the falls sub‐dimension. However, literature suggests that falls are a significant source of medical errors [31, 39]. Contributing factors may include the relatively lower experience of younger nurses, insufficient education, intense and challenging shifts and an inadequate awareness of their physical capabilities and fall risks.

Nurses working in paediatric wards had lower mean scores in PMMS and demonstrated a higher tendency for medical errors compared to those in other wards. This finding is consistent with similar research, which reported that nurses in paediatric wards were more prone to medical errors related to patient and material safety [40]. Prior studies have highlighted that the environment of paediatric wards, including high external stimuli and challenging conditions, contributes to the increased risk of medical errors [22, 40].

Furthermore, the study found that the mean score for the falls sub‐dimension was lower in nurses working in paediatric wards compared to those in other clinics, with a higher likelihood of medical errors. This is consistent with previous research, which indicated a higher tendency for falls‐related medical errors among paediatric ward nurses [40]. This may be attributed to factors such as the mobility of paediatric patients, differing care needs, a high patient‐to‐nurse ratio, and the challenging nature of paediatric care.

## 5 Limitations

The study has several limitations. First, the data were collected from nurses working in the paediatric wards of a hospital providing tertiary healthcare services and were based on the nurses' self‐reports. This limits the generalizability of the findings. Additionally, since the data were collected while the nurses were at work, factors such as work fatigue, the intensity of the service and the number of patients may have influenced their responses, further constraining the generalizability of the study.

## 6 Conclusion

The study revealed that nurses working in paediatric clinics experience moderate inertia, with their propensity for medical errors generally being low. However, this inertia may negatively impact their performance and work efficiency. To address this, it is essential to prevent inertia through organized training, programmes, congresses, and activities aimed at updating care approaches and implementing evidence‐based nursing practices.

It has been observed that nurses in paediatric wards show increased tendencies for errors related to patient monitoring, material safety and falls. To mitigate these issues, strategic approaches are crucial. Setting clear goals and planning effectively can help nurses organize their tasks. Prioritizing workload through daily, weekly and monthly plans can aid in completing tasks efficiently and on time. Identifying sources of motivation and creating support networks among colleagues can enhance work motivation. Regular physical activity can boost mental alertness by increasing energy levels, while maintaining a distraction‐free work environment can improve focus and performance. Celebrating small successes and viewing mistakes as learning opportunities can help maintain high morale and motivation.

These findings highlight the need for improvements in nursing practices. It is evident that increasing training and development opportunities, reviewing working conditions and considering socio‐demographic factors are necessary. Targeted strategies and additional training should be planned and implemented to enhance patient safety, particularly in paediatric clinics. Further comprehensive research is needed to improve nursing service quality and reduce medical errors.

## Conflicts of Interest

The authors declare no conflicts of interest.

## Data Availability

The data that support the findings of this study are available from the corresponding author upon reasonable request.
